# Action of Gelseminum Senpervirens

**Published:** 1877-06

**Authors:** 


Action of Gelseminum Senpervirens.—Dr. Sidney Ringer and Mr. Wm. Murrell have published (*Lancet*, Oct. 21, 1876) the results of a series of experiments made by them to ascertain the influence of gelseminum on the circulation. These experiments they say show:—

"1. That gelseminum produces but little, if any, effect on the pulse.

"2. That it does not affect the blood pressure.

"3. That in man it probably acts on the respiratory centre less energetically than in the lower animals.

"4. That in man it acts on the muscles of the eye, and produces other symptoms before it influences the respiratory centre.

"5. That in man it, in all probability, affects the spinal cord before the respiratory centre.

"6. That it exerts no influence on the mind, and none on the cutaneous sensibility.

"7. That it does not affect the temperature."
—*Am. Jour. Med. Sciences.*

